# Genetic evidence for a potential role of cytoskeleton-associated protein 2 in lung squamous cell carcinoma: a bidirectional mendelian randomization study

**DOI:** 10.1186/s13019-026-04634-4

**Published:** 2026-07-19

**Authors:** Qiang Xie, Yuzuo Lin, Qingtong Wu, Ziyan Lin, Yanhong Cheng, Guodong Zhong, Lei Wang

**Affiliations:** 1 Department of Pathology, Fujian Province Second People’s Hospital, The Second Affiliated Hospital of Fujian University of Traditional Chinese Medicine, Fuzhou, 350000 Fujian province China; 2 Union College of Clinical Medicine, Fujian Medical University Union Hospital, Fuzhou, 350000 Fujian province China; 3 Department of Cardiovascular Surgery, Fujian Medical University Union Hospital, Fuzhou, 350000 Fujian province China; 4 Engineering Research Center of Tissue and Organ Regeneration, Fujian Province University, Fuzhou, 350000 Fujian province China; 5 No. 282, Wusi Road, Gulou District, Fuzhou city, Fujian Province China; 6 Minhou County, No. 1, Xueyuan Road, Shangjie Town, Fuzhou city, Fujian Province China; 7 No. 29, Xinquan Road, Gulou District, Fuzhou city, Fujian Province China

**Keywords:** CKAP2, Lung cancer, Mendelian randomization

## Abstract

**Background:**

Cytoskeleton-associated protein 2 (CKAP2) plays a role in tumor proliferation, but its causal relationship with lung cancer remains unclear. This study employed a bidirectional two-sample Mendelian randomization (MR) analysis to investigate this association.

**Methods:**

Using genome-wide association and FinnGen data, we assessed the potential causal relationship between genetically predicted plasma CKAP2 levels and lung cancer risk, including its subtypes. The inverse-variance weighted (IVW) method served as the primary analysis, supplemented by MR-Egger and weighted median. Sensitivity analyses included leave-one-out, Cochran’s Q, MR-Egger intercept, and MR-PRESSO. To assess generalizability, we replicated the forward MR analysis using an independent deCODE pQTL dataset.

**Results:**

Forward MR showed a nominal association between elevated CKAP2 and lung squamous cell carcinoma (LUSC) risk (IVW: OR = 1.18; 95% CI = 1.01–1.38; *P* = 0.033). However, this did not survive Bonferroni correction (α = 0.0125) and was not replicated in an independent deCODE dataset (OR = 0.875; *P* = 0.493). No significant associations were found for overall lung cancer, lung adenocarcinoma, or small cell lung cancer. Sensitivity analyses revealed no heterogeneity or horizontal pleiotropy. Reverse MR found no causal effect of lung cancer on CKAP2 levels.

**Conclusions:**

This study provides nominally significant genetic evidence for an association between elevated CKAP2 levels and LUSC risk, but the finding does not survive multiple-testing correction and was not replicated in an independent dataset. Further validation in larger cohorts with harmonized proteomic platforms is required before CKAP2 can be considered a therapeutic target.

**Supplementary Information:**

The online version contains supplementary material available at https://doi.org/10.1186/s13019-026-04634-4.

## Background

### Disease burden and heterogeneity of lung cancer

Lung cancer remains one of the leading causes of cancer-related mortality globally, representing a significant public health challenge. In 2022, approximately 2.5 million new cases were diagnosed worldwide, resulting in about 1.8 million deaths, which accounts for 18.7% of all cancer-related fatalities [1]. Histologically, lung cancer is classified into small cell lung cancer (SCLC) and non-small cell lung cancer (NSCLC), with the latter encompassing major subtypes such as lung adenocarcinoma (LUAD) and lung squamous cell carcinoma (LUSC) [2]. These subtypes exhibit distinct pathogenic mechanisms that contribute to considerable heterogeneity in clinical behavior, prognosis, and treatment responses [3]. Over the past decade, advancements in low-dose computed tomography (CT) screening, biomarker-guided targeted therapies, and immunotherapy have collectively led to a 4.5%-point improvement in the five-year survival rate [4]. Despite these advancements, overall prognosis remains unfavorable; the five-year survival rate stands at merely 18.6% [1]. Tobacco smoking constitutes the primary environmental risk factor for lung cancer, accounting for approximately 85% of related fatalities worldwide—including those associated with exposure to secondhand smoke [5]. Previous studies indicate that a positive family history increases the risk of developing lung cancer by a factor of 1.7 [6]. However, the role of genetic factors in susceptibility to lung cancer remains inadequately understood.

### Biological significance of cytoskeleton-associated protein 2 and research gaps

Cytoskeleton-associated protein 2 (CKAP2), located on chromosome 13q14.3, is a microtubule-associated protein that plays a crucial role in regulating microtubule stability and cell division through phosphorylation mechanisms. It is integral to mitosis and the dynamics of microtubules [7–9]. A deficiency in CKAP2 can lead to abnormal mitotic events, such as the formation of multipolar spindles and various mitotic defects, which contribute to aneuploidy and genomic instability [10]. CKAP2 has been identified as a direct transcriptional target of TP53 and participates in a p53-dependent positive feedback loop controlling cell cycle progression and apoptosis [11]. Clinical studies have documented CKAP2 dysregulation across multiple cancer types. In cervical cancer, CKAP2 modulates the tumor microenvironment by promoting pro-tumor macrophage infiltration and angiogenesis via NF-κB signaling [12]. In breast cancer, elevated levels of CKAP2 expression are significantly correlated with decreased overall survival rates as well as reduced relapse-free survival; additionally, it enhances invasiveness by facilitating epithelial-mesenchymal transition [13, 14]. The homologous protein cytoskeleton associated protein 2 like (CKAP2L) has been shown to promote NSCLC progression by regulating RNA polymerase II-mediated transcriptional elongation; its overexpression correlates with increased tumor invasion, lymph node metastasis, and poor prognosis [15, 16]. Despite these observational links, the evidence base for CKAP2 in cancer has been correlational rather than causal.

### Application of MR in etiological research of lung cancer

Mendelian randomization (MR) leverages randomly assorted genetic variants as instrumental variables to mimic the design of a randomized controlled trial, thereby mitigating confounding and reverse causation [17]. To date, although MR methods may have been applied in genome- or proteome-wide studies of lung cancer, no significant causal association has been identified between CKAP2 and lung cancer. In the present study, we conducted a bidirectional two-sample MR analysis integrating plasma pQTL data with lung cancer Genome-Wide Association Study (GWAS) outcomes to assess whether genetically predicted CKAP2 levels influence lung cancer risk, and reciprocally, whether lung cancer genetic liability influences CKAP2 levels. We complemented the MR analysis with transcriptomic and proteomic profiling from TCGA and CPTAC to contextualize CKAP2 expression, immune correlates, and drug sensitivity patterns in LUSC.

## Materials and methods

### Study design

**Fig. 1:**
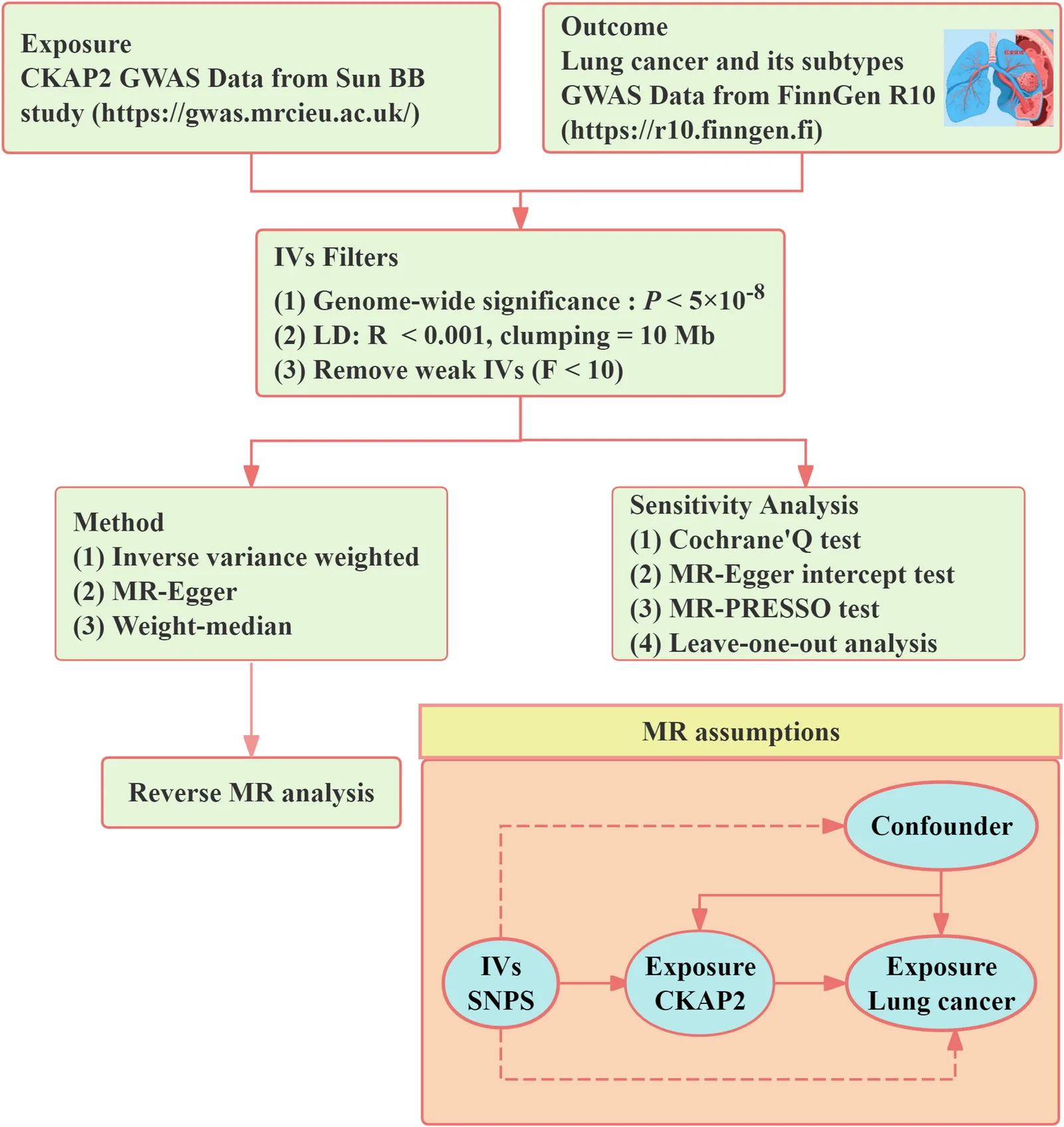
The overall design of the study. MR, Mendelian randomization; CKAP2, Cytoskeleton-associated protein 2; GWAS, Genome-Wide Association Study; IVs, instrumental variables; LD, linkage disequilibrium; SNPs, Single nucleotide polymorphisms

In accordance with Mendel’s law of inheritance, the random assortment of genes during meiosis resembles a randomized controlled experiment, effectively minimizing the influence of confounding factors. This study employed a bidirectional two-sample MR design (Fig. 1). The forward MR analysis evaluated the causal effect of genetically predicted plasma CKAP2 protein levels on lung cancer and its subtypes (LUSC, LUAD, SCLC). The reverse MR analysis assessed whether genetic liability to lung cancer influences CKAP2 protein levels. The MR analysis was conducted based on three fundamental assumptions: (1) Relevance – the genetic variants selected as instrumental variables (IVs) must be robustly associated with the exposure (CKAP2 protein levels); (2) Independence – the IVs must be independent of any confounders that influence both the exposure and the outcome; and (3) Exclusion restriction – the IVs must affect the outcome (lung cancer) exclusively through the exposure, with no alternative pathways (horizontal pleiotropy) [17].

### Data sources

Summary data for CKAP2 protein quantitative trait loci (pQTLs) were obtained from the GWAS dataset (https://gwas.mrcieu.ac.uk/). Single nucleotide polymorphisms (SNPs) demonstrating strong associations with CKAP2 protein levels were selected as IVs. The exposure data originated from a study conducted by Sun et al. (GWAS ID: prot-a-564), which quantified plasma CKAP2 levels using a proteomic chip in a cohort of 3301 individuals of European descent [17] (Table 1). Genetic association data for the outcome variables, specifically lung cancer and its subtypes, were sourced from the FinnGen consortium release dated December 18, 2023. The FinnGen study represents a large-scale genomic initiative that integrates genetic and health information from over 500,000 Finnish biobank participants, aiming to elucidate the genetic underpinnings of diseases and their susceptibility [18]. Independent pQTL data were obtained from the deCODE study [19], which measured plasma proteins using the SomaScan platform in 35,559 Icelandic individuals. As all original studies received approval from their respective institutional review boards, no additional ethical clearance or participant consent was required for this analysis. Detailed descriptions of both the exposure and outcome datasets are summarized in Table 1.

**Table 1:** Characteristics of exposure and outcome variables

	Exposures/Outcomes	GWAS ID	Data source	Sample size	Cases/ Controls
Exposures	Cytoskeleton-associated protein 2 (CKAP2)	prot-a-564	Sun BB et al. [17]	3301	-
Outcomes	Total lung cancer	finngen_R10_C3_BRONCHUS_LUNG_EXALLC	FinnGen [18]	320,533	6340/314,193
Outcomes	Lung adenocarcinoma (LUAD)	finngen_R10_C3_NSCLC_ADENO_EXALLC	FinnGen [18]	315,783	1590/314,193
Outcomes	Lung squamous cell carcinoma (LUSC)	finngen_R10_C3_NSCLC_SQUAM_EXALLC	FinnGen [18]	315,703	1510/314,193
Outcomes	Small cell lung cancer (SCLC)	finngen_R10_C3_SCLC_EXALLC	FinnGen [18]	314,910	717/314,193

GWAS, Genome-Wide Association Study

### Selection of IVs for CKAP2 protein levels

MR employs genetic variants as IVs to evaluate causal effects of risk factors on outcomes [20]. In all two-sample MR analyses, SNPs were selected at a genome-wide significance threshold of *P* < 5 × 10⁻⁸. For reverse MR analysis, a less stringent threshold of *P* < 5 × 10⁻⁶ was applied [21], as fewer than two SNPs for LUSC and LUAD reached the conventional threshold in the reverse GWAS summary statistics. To minimize linkage disequilibrium (LD), SNPs were pruned using a clumping window of < 10,000 kb and R² < 0.001. The strength of IVs was assessed using the F-statistic: F = [R² × (N − K − 1)] / [K × (1 − R²)], where R² denotes the cumulative variance explained by the SNPs for the exposure, N the sample size of the exposure dataset, and K the number of SNPs included. An F-statistic > 10 indicates a sufficiently robust IV to mitigate weak instrument bias [22]. SNPs with an F-statistic below this threshold were excluded. Ultimately, six SNPs related to CKAP2 protein were included in the final analysis (Supplementary Table S1), with F-statistics ranging from 30.52 to 173.87—all meeting the criteria for valid IVs.

### MR analysis

In the forward MR analysis, we assessed the causal effect of CKAP2 protein levels on lung cancer using three distinct methods. The primary approach was the random-effects inverse-variance weighted (IVW) method, supplemented by MR-Egger and weighted median methods [23, 24]. Effect sizes were expressed as odds ratios (OR) with 95% confidence intervals (CI), representing the change in lung cancer risk per one-standard deviation (SD) increase in genetically predicted CKAP2 protein levels.

Results were visualized using the R package “TwoSampleMR,” which generated scatter, forest, and funnel plots, with emphasis on the IVW estimates. In scatter plots, a small intercept indicates limited confounding, supporting the robustness of the findings; a positive slope suggests a risk factor, while a negative slope implies a protective effect. Forest plots assessed the contribution of individual SNPs: points clustered on the left signify protective effects, and those on the right represent risk effects. Funnel plots evaluated symmetry—a symmetric distribution of IVs around the IVW line is consistent with no directional pleiotropy, in accordance with the second assumption of MR.

Similarly, reverse MR analysis was performed to examine whether genetic predisposition to lung cancer influences CKAP2 protein levels, employing the same three methods (IVW, MR-Egger, and weighted median). All analyses were conducted using the “TwoSampleMR” (version 0.6.8) and “MR-PRESSO” (version 1.0) R packages in R version 4.4.2. Results are presented as OR with 95% CI, and a two-sided *P*-value < 0.05 was considered nominally significant for the primary IVW estimates. To account for multiple testing across the four forward MR outcomes (overall lung cancer, LUSC, LUAD, and SCLC), we applied a Bonferroni-corrected threshold of α = 0.0125 (0.05/4). Results with *P* < 0.05 but ≥ 0.0125 were considered suggestive associations rather than definitive conclusions.

### Sensitivity analysis

Sensitivity analyses were performed to evaluate the robustness of the MR estimates, including leave-one-out analysis, heterogeneity assessment, and pleiotropy evaluation. In the leave-one-out analysis, each SNP was iteratively excluded and the effect estimate recalculated from the remaining SNPs. If removal of a specific SNP substantially altered the causal estimate, it was considered influential and excluded from the final analysis. Heterogeneity among IVs was assessed using Cochran’s Q test under both IVW and MR-Egger methods; a *P*-value > 0.05 indicated no substantial heterogeneity. Horizontal pleiotropy was evaluated through the MR-Egger intercept and the MR-Pleiotropy RESidual Sum and Outlier (MR-PRESSO) global test. A non-significant MR-Egger intercept (*P* > 0.05) or a non-significant MR-PRESSO global test (*P* > 0.05) argued against the presence of horizontal pleiotropy, supporting the validity of the instrumental variable assumptions.

### Analysis of CKAP2 mRNA expression and functional profiling in LUSC

To investigate the relationship between CKAP2 expression and clinicopathological features in LUSC, we evaluated differences in expression levels based on patient sex, age, and tumor stage using the Wilcoxon rank-sum test within the LUSC cohort. Associations between CKAP2 mRNA expression and clinicopathological variables were assessed using Pearson correlation analysis. The Cancer Genome Atlas (TCGA) [25] is a publicly accessible repository that provides comprehensive, multi‑dimensional genomic, transcriptomic, and clinical data for over 30 human cancer types. We utilized the TCGA‑LUSC cohort, which comprises RNA‑sequencing data and corresponding clinical annotations from 501 primary LUSC tumors and 51 adjacent normal lung tissues. The ‘TCGAplot’ R package was utilized to analyze this dataset [26]. Initially, Student’s t‑test was employed to compare the expression levels of CKAP2 mRNA between LUSC tissues and adjacent normal tissues. Following this, a receiver operating characteristic (ROC) curve was generated, and the area under the curve (AUC) was calculated to evaluate the diagnostic potential of CKAP2 mRNA in differentiating LUSC from normal tissues. Given that CKAP2 is a direct transcriptional target of p53 [11], we further examined the correlation between CKAP2 and TP53 mRNA expression.

To explore the biological function of CKAP2, Pearson correlation analysis was performed between CKAP2 expression and all transcriptome-wide genes. The top 20 positively and negatively correlated genes were selected for co-expression network construction. Gene Ontology (GO) enrichment analysis was conducted using the clusterProfiler package with Benjamini-Hochberg correction.

### Association of CKAP2 protein expression with drug sensitivity and immune microenvironment in LUSC

The Clinical Proteomic Tumor Analysis Consortium (CPTAC) is a National Cancer Institute effort applying large-scale proteome and genome analyses (proteogenomics) to advance the understanding of the molecular basis of cancer [27]. All mass spectrometry-based proteomic, genomic, and imaging data are publicly available through the CPTAC Proteomic Data Commons (https://pdc.cancer.gov/). For the present analysis, we used the CPTAC-LUSC discovery cohort, comprising proteomic data from 110 primary tumors and 102 adjacent normal lung tissues. Protein abundance was quantified using mass spectrometry-based Tandem Mass Tag technology. Systematic analyses were conducted using the ProteoCancer Analysis Suite R package (version 0.1.0) [28], a tool developed to facilitate integrative analysis of CPTAC proteomics, phosphoproteomics, and transcriptomics data. CKAP2 protein differential expression was assessed by Student’s t-test, and CKAP2–TP53 protein correlation was evaluated using Pearson correlation.

CKAP2 has been reported to modulate the tumor microenvironment in cervical cancer by promoting pro-tumor M2 macrophage infiltration and enhancing angiogenesis via NF-κB signaling [12]. To explore whether CKAP2 plays a similar role in LUSC, we examined the association between CKAP2 protein expression and immune cell infiltration levels using the TIMER algorithm in the CPTAC-LUSC cohort.

Additionally, given CKAP2’s function as a microtubule-stabilizing protein and its prior association with paclitaxel sensitivity in cervical cancer [12], we assessed correlations between CKAP2 expression and drug sensitivity using z-scores from the Cancer Therapeutics Response Portal, where higher scores indicate greater drug resistance. These sensitivity scores are positively correlated with the half-maximal inhibitory concentration (IC₅₀); thus, higher scores correspond to increased resistance. All correlations—CKAP2 versus TP53, immune infiltration, and drug sensitivity—were evaluated using Pearson correlation.

### Cross-dataset comparison analysis in an independent pQTL dataset

To assess the generalizability of our findings, we replicated the forward MR analysis using the independent deCODE pQTL dataset [19] (*N* = 35559 Icelandic individuals). The same IV selection criteria and MR analytical methods were applied, and FinnGen R10 LUSC data were used as the outcome for consistency in phenotype definition.

## Results

### Potential relationship between CKAP2 and lung cancer and its subtypes

**Fig. 2:**
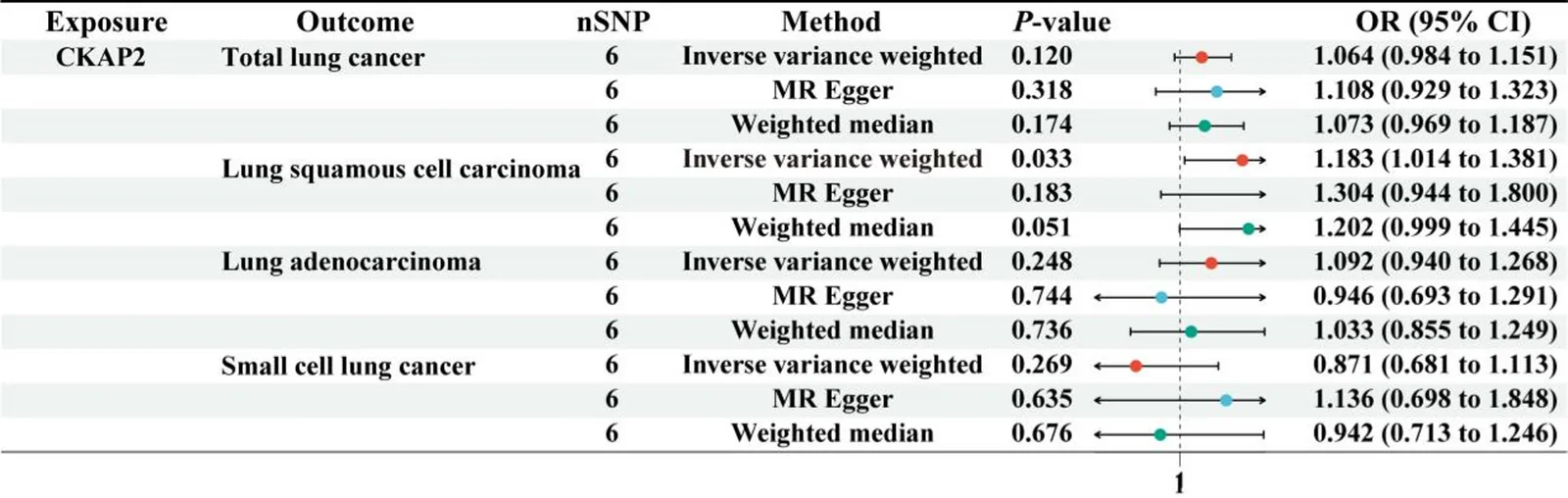
Forest plots illustrating the results of MR analysis for CKAP2 expression in relation to lung cancer and its subtypes. Odds ratios along with 95% confidence intervals were calculated utilizing three distinct methodologies. MR, Mendelian randomization; CKAP2, Cytoskeleton-associated protein 2; SNP, single nucleotide polymorphisms; CI, confidence interval; OR, odds ratio

MR analysis using the IVW method revealed that elevated CKAP2 expression was a nominally significant risk factor for LUSC (OR = 1.18, 95% CI: 1.01–1.38, *P* = 0.033). The weighted median method yielded directionally consistent estimates of borderline statistical significance (OR = 1.20, 95% CI: 1.00–1.45, *P* = 0.051). Consistently, the forest plot showed that most individual SNPs exhibited effect estimates on the risk-increasing side (OR > 1), further supporting CKAP2 as a potential risk factor for LUSC (Fig. 2). In contrast, no significant associations were observed between CKAP2 and overall lung cancer or other histological subtypes.

**Fig. 3:**
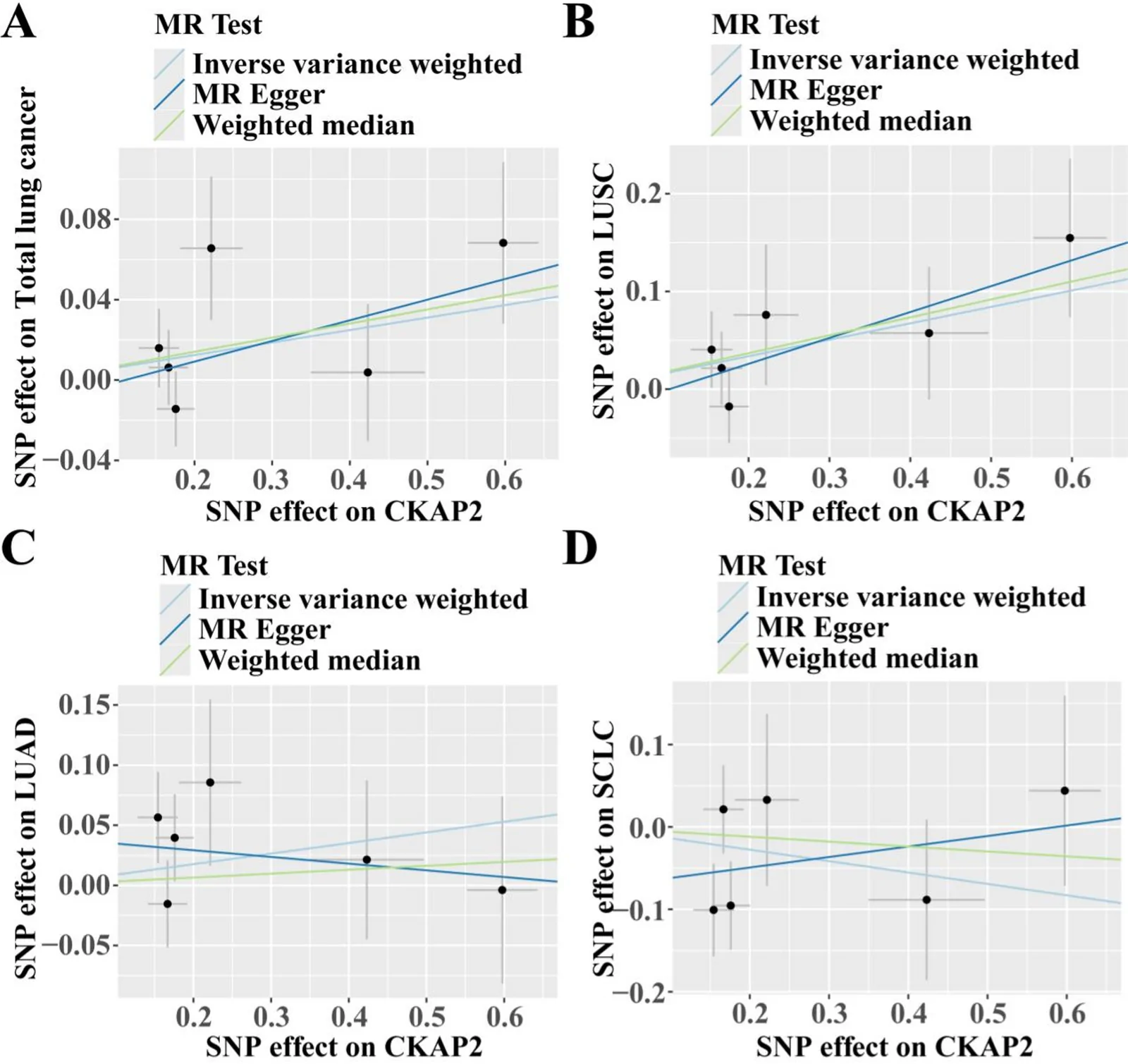
Scatter plot showing the Mendelian randomization analysis of CKAP2 expression with (**A**) total lung cancer, (**B**) LUSC, (**C**) LUAD, and (**D**) SCLC. Each point represents an individual SNP. The slopes of the lines represent the causal estimates from different MR methods (IVW, MR-Egger, weighted median)

**Fig. 4:**
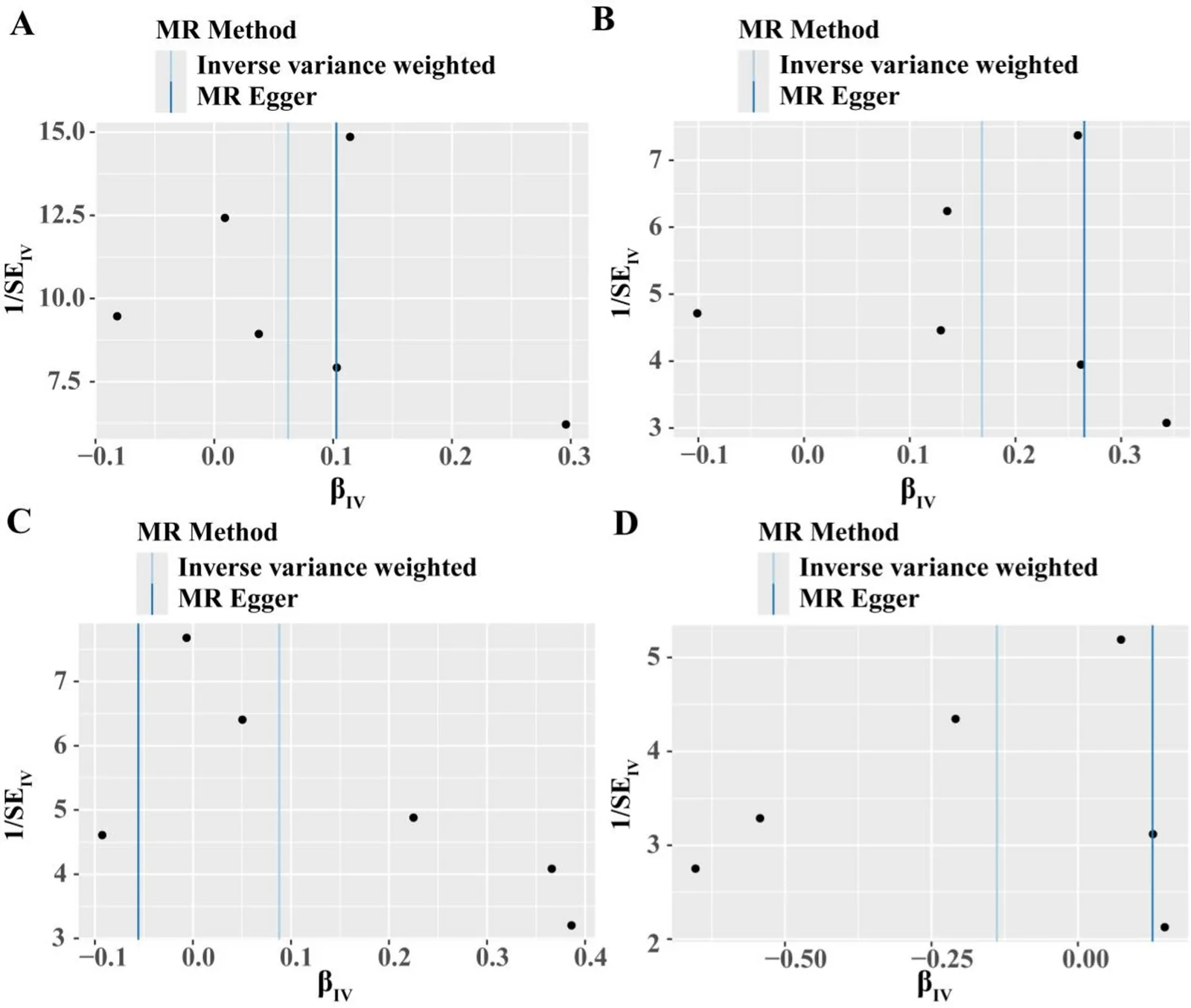
Funnel plots of MR analysis for CKAP2 expression in relation to lung cancer and its subtypes. (**A**) Funnel plots of MR analysis examining the association between CKAP2 expression and (**A**) total lung cancer, (**B**) LUSC, (**C**) LUAD, and (**D**) SCLC. MR, Mendelian randomization; CKAP2, cytoskeleton-associated protein 2; SE: standard error

The scatter plot showed a positive slope in the fitted line, indicating a risk-increasing effect of CKAP2 on LUSC; the near-zero intercept suggested negligible horizontal pleiotropy, supporting the validity of the IVs (Fig. 3). The funnel plot showed approximate symmetry, suggesting no substantial publication bias and reinforcing the stability of the MR estimates (Fig. 4).

However, under the more stringent Bonferroni-corrected threshold (α = 0.0125), this association (*P*=0.033) did not reach statistical significance and should therefore be interpreted as suggestive rather than definitive.

### Causal effect of lung cancer and its subtypes on CKAP2 expression

**Fig. 5:**
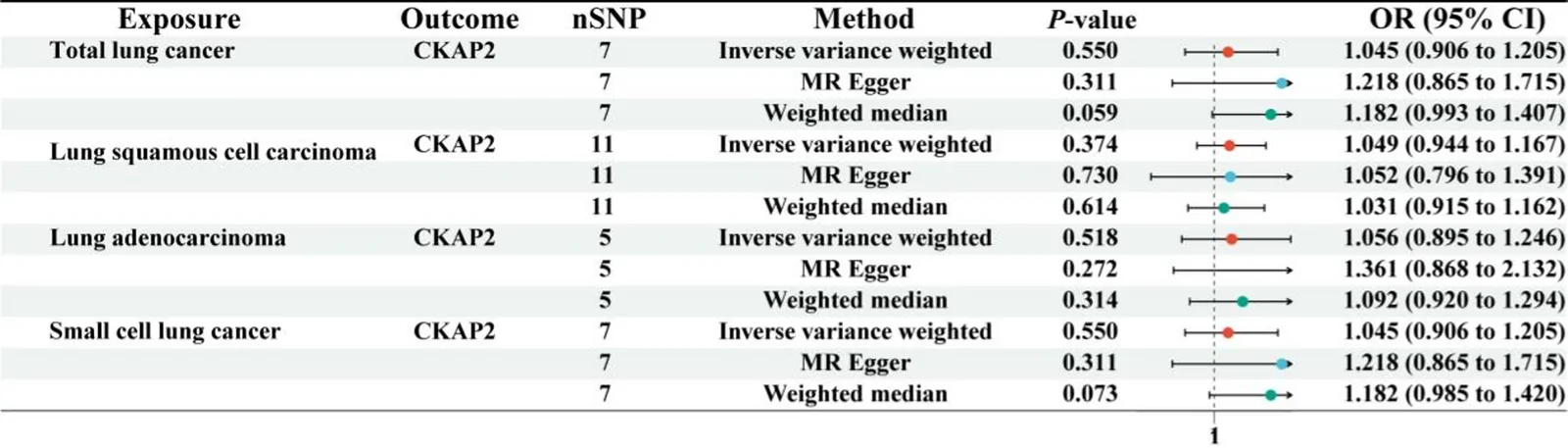
Forest plot illustrating the reverse MR analysis that assesses the causal relationship between lung cancer and its subtypes on CKAP2 expression. Odds ratios along with 95% confidence intervals were calculated utilizing three distinct methodologies. MR, Mendelian randomization; CKAP2, cytoskeleton-associated protein 2; SNP, single nucleotide polymorphisms; CI, confidence Interval; OR, odds ratio

A reverse MR analysis was performed to investigate whether genetic predisposition to lung cancer and its subtypes has a potential causal impact on plasma CKAP2 protein levels. The results revealed no significant causal association between the genetically predicted risk of lung cancer (or its subtypes) and plasma CKAP2 levels, as assessed by any of the three analytical methods employed: IVW, MR-Egger regression, or weighted median (All *P* > 0.05; Fig. 5).

### Sensitivity analysis

**Fig. 6:**
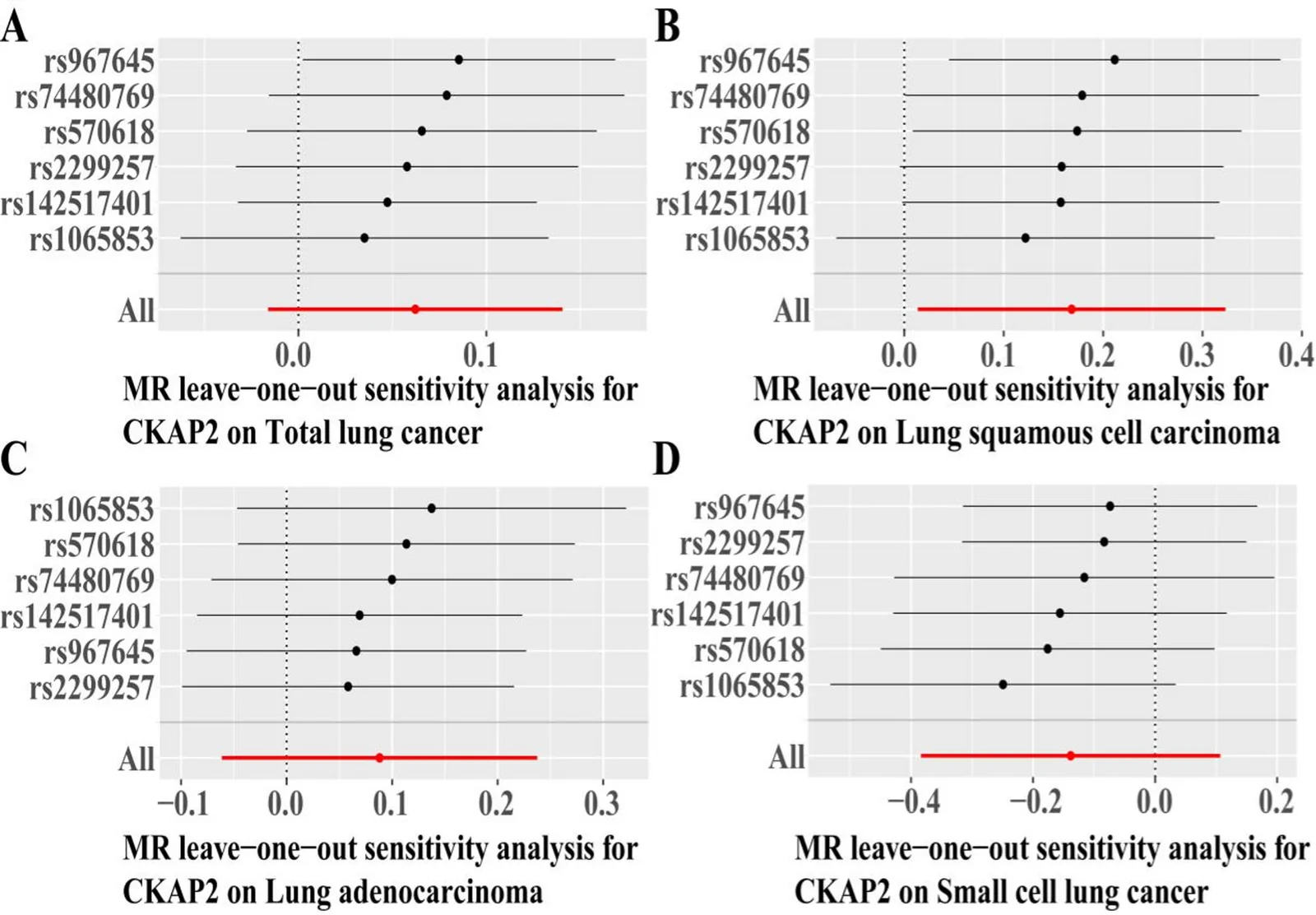
Leave-one-out sensitivity analysis of the causal effects of CKAP2 on lung cancer and its subtypes. (**A**) Effect on total lung cancer. (**B**) Effect on lung squamous cell carcinoma. (**C**) Effect on lung adenocarcinoma. (**D**) Effect on small cell lung cancer. MR, Mendelian randomization; CKAP2, cytoskeleton-associated protein 2; SNPs, single nucleotide polymorphisms

Following the primary MR analysis, sensitivity analyses were conducted to assess the robustness of the causal estimates. The leave-one-out analysis demonstrated that the confidence intervals consistently remained on the same side of the null line with each iterative exclusion of individual SNPs. This finding indicates that no single SNP disproportionately influenced the overall association. Such results support the absence of influential outliers and further reinforce the stability of the inferred causal relationship between CKAP2 and lung cancer risk, as well as its subtypes (Fig. 6).

Cochran’s Q test indicated no significant heterogeneity in the causal effect of CKAP2 on lung cancer and its subtypes, as evidenced by *P*-values exceeding 0.05 for both the IVW and MR-Egger methods. The absence of horizontal pleiotropy was further corroborated by a non-significant intercept in the MR-Egger regression (*P* > 0.05) and by the results of the MR-PRESSO Global Test (*P* > 0.05). These findings reinforce the robustness of the potential causal association between CKAP2 and lung cancer, including its various subtypes (Table 2).

**Table 2:** Heterogeneity and horizontal pleiotropy tests for the potential causal association of CKAP2 with lung cancer and its subtypes

Exposure	Outcomes	Heterogeneity test				Pleiotropy test			
		IVW		MR-Egger		MR-Egger			MR-PRESSO Global test *P*
		Q-statistics	*P*	Q-statistics	*P*	Intercept	SE	*P*	
CKAP2	Total lung cancer	5.15	0.40	4.84	0.30	-0.01	0.02	0.63	0.46
CKAP2	Lung squamous cell carcinoma	2.56	0.77	2.11	0.72	-0.03	0.04	0.54	0.76
CKAP2	Lung adenocarcinoma	3.92	0.56	2.86	0.58	0.04	0.04	0.36	0.59
CKAP2	Small cell lung cancer	6.13	0.29	4.47	0.35	-0.07	0.06	0.29	0.34

*P *> 0.05 indicates no significant pleiotropy; MR, Mendelian randomization; CKAP2, Cytoskeleton-associated protein 2; IVW, inverse-variance weighted; PRESSO, Pleiotropy RESidual Sum and Outlier; SE, standard error

### The Association between CKAP2 mRNA Expression Levels and Clinical Significance in LUSC

**Fig. 7:**
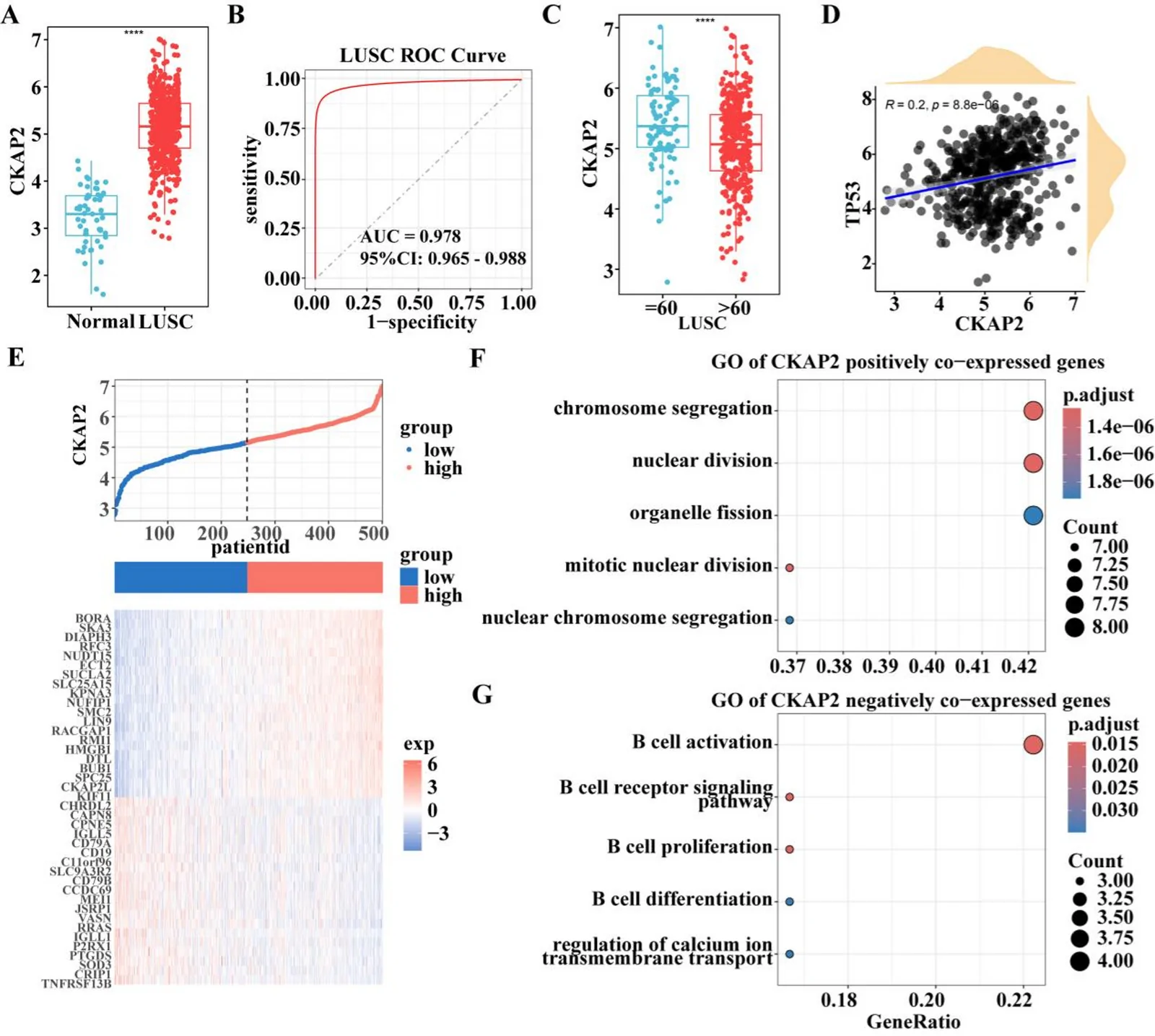
The association between CKAP2 mRNA expression levels and clinical significance in LUSC. (**A**) Comparison of CKAP2 expression levels in normal tissues versus LUSC tissues. (**B**) ROC curve analysis assessing the diagnostic value of CKAP2 for LUSC. (**C**) Analysis of CKAP2 mRNA expression among LUSC patients categorized by different age groups. (**D**) Examination of the correlation between CKAP2 and TP53 expression in LUSC tissues. (**E**) Risk stratification of LUSC samples based on CKAP2 expression, accompanied by a heatmap illustrating the top 20 genes that are positively and negatively correlated with CKAP2. (**F**) Identification of biological processes enriched among genes co-expressed with CKAP2. (**G**) Identification of biological processes enriched among genes expressed antagonistically to CKAP2. CKAP2, cytoskeleton-associated protein 2; LUSC, lung squamous cell carcinoma; ROC, receiver operating characteristic; GO, Gene Ontology

Analysis of TCGA data demonstrated significant upregulation of CKAP2 mRNA in LUSC tumor tissues (*n* = 501) compared to normal lung samples (*n* = 51; *P* < 0.001; Fig. 7A). ROC curve analysis revealed robust diagnostic performance, with an AUC of 0.978 (95% CI: 0.965–0.988) for distinguishing LUSC from non-cancerous tissues (Fig. 7B).

CKAP2 mRNA expression was significantly higher in LUSC patients over 60 years (*n* = 376) than in those under 60 (*n* = 91, *P* < 0.001; Fig. 7C). No significant associations were found with sex or pathological tumor stage (Supplementary Figure S1). Correlation analysis further revealed a significant co-expression relationship between CKAP2 and the tumor suppressor gene TP53 in LUSC tissues (Fig. 7D). Although the correlation was weak (Pearson *r* = 0.2), it was highly statistically significant (*P* < 0.001).

LUSC samples were dichotomized into low- and high-expression groups based on the median CKAP2 mRNA expression value (log₂(TPM + 1) = 5.16). The top 20 genes most significantly positively and negatively correlated with CKAP2 were selected and presented in a heatmap (Fig. 7E). The top five positively correlated genes included BORA, SKA3, DIAPH3, RFC3, and NUDT15; the top five negatively correlated genes included CHRDL2, CAPN8, CPNE5, IGLL5, and CD79A. The complete list is provided in Supplementary Table S2. The biological functions enriched among positively correlated genes (indicating cooperative roles) and negatively correlated genes (indicating antagonistic roles) are illustrated in Fig. 7F and G, respectively. Positively correlated genes were primarily associated with chromosome segregation, nuclear division, organelle fission, mitotic nuclear division, and nuclear chromosome segregation. Negatively correlated genes were predominantly involved in B cell activation, B cell receptor signaling pathways, B cell proliferation, B cell differentiation, and regulation of calcium ion transmembrane transport.

### Protein expression levels of CKAP2 in LUSC and its association with chemotherapeutic drug sensitivity

**Fig. 8:**
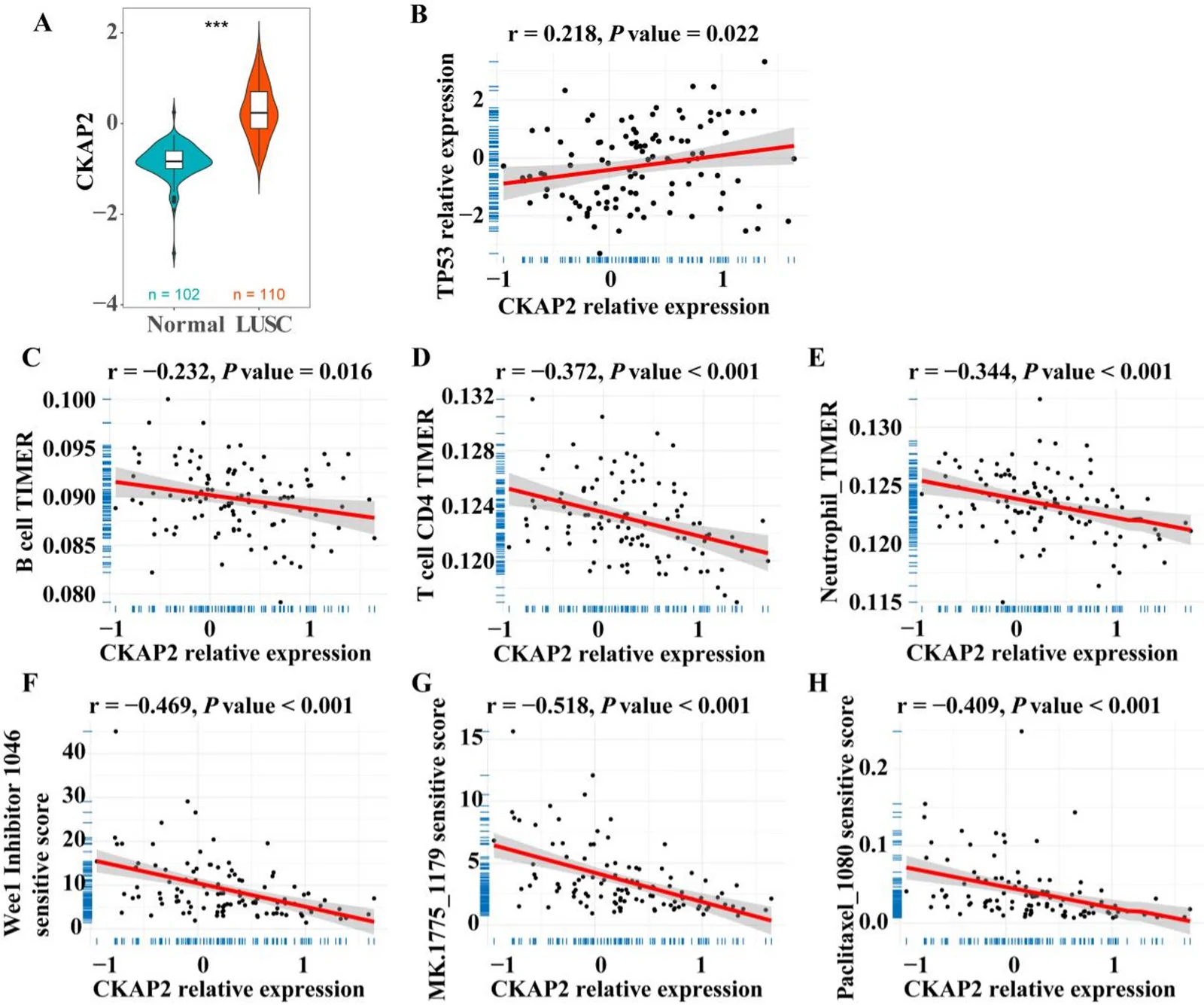
CKAP2 expression in LUSC: protein levels, immune infiltration, and drug sensitivity. (**A**) Comparison of CKAP2 protein levels between LUSC and normal tissues. (**B-H**) Scatter plots showing correlations of CKAP2 expression with TP53 (**B**), B cells (**C**), CD4⁺ T cells (**D**), neutrophils (**E**), Wee1 inhibitor (ID:1046) (**F**), MK-1775 (ID:1179) (**G**), and Paclitaxel (ID:1080) (**H**). CKAP2, cytoskeleton-associated protein 2; LUSC, lung squamous cell carcinoma

Analysis of the CPTAC-LUSC dataset revealed significantly higher CKAP2 protein expression in tumor tissues (*n* = 110) compared to normal lung samples (*n* = 102; *P* < 0.001; Fig. 8A). Correlation analysis further showed a positive association between CKAP2 and TP53 protein expression (*r* = 0.218, *P* < 0.05; Fig. 8B).

Elevated CKAP2 expression was also closely associated with alterations in the tumor immune microenvironment. Specifically, CKAP2 levels showed significant negative correlations with infiltration of B cells (*r* = − 0.232, *P* < 0.05; Fig. 8C), CD4⁺ T cells (*r* = − 0.372, *P* < 0.001; Fig. 8D), and neutrophils (*r* = − 0.344, *P* < 0.001; Fig. 8E).

Furthermore, elevated CKAP2 expression was associated with increased sensitivity to several targeted agents, including a Wee1 inhibitor (ID: 1046; *r* = − 0.469, *P* < 0.001; Fig. 8F), MK-1775 (ID: 1179; *r* = − 0.518, *P* < 0.001; Fig. 8G), and paclitaxel (ID: 1080; *r* = − 0.409, *P* < 0.001; Fig. 8H). These findings suggest that CKAP2 may serve as a potential biomarker for predicting chemotherapeutic response in LUSC.

### Replication in an independent dataset

The replication results are presented in Supplementary Table S3. The results did not confirm the suggestive association observed in the primary Sun et al. dataset. In the deCODE cohort, genetically predicted CKAP2 levels showed no significant association with LUSC risk (IVW: OR = 0.875, 95% CI: 0.598–1.281, *P* = 0.493). Notably, the direction of effect was opposite to that observed in the discovery analysis (OR = 0.875 vs. OR = 1.18), indicating substantial between-dataset heterogeneity.

## Discussion

In this study, we performed a univariable bidirectional two-sample MR analysis to evaluate the causal relationship between circulating CKAP2 levels and lung cancer. Our findings suggest that genetic predisposition to higher circulating CKAP2 levels may be associated with an increased risk of LUSC, but not with overall lung cancer, LUAD, or SCLC. However, several critical limitations preclude any definitive causal interpretation of these findings. First, the observed *P*-value (0.033) did not survive correction for multiple testing across the four lung cancer outcomes evaluated (Bonferroni-adjusted α = 0.0125). Second, our attempt to validate the primary finding in an independent deCODE pQTL dataset not only failed to replicate the association but also revealed an opposite direction of effect. This discordance substantially undermines confidence in the robustness of the reported association. Consequently, the MR findings should be interpreted as hypothesis-generating rather than confirmatory evidence for a causal role of CKAP2 in LUSC tumorigenesis.

To exclude the potential interference of reverse causation, we further performed a reverse MR analysis using the same instrument selection criteria as the forward analysis. The null findings from this analysis indicate that genetic susceptibility to lung cancer does not significantly influence circulating CKAP2 levels within the limits of our statistical power. Moreover, the consistent estimates across all three MR methods argue against bias from horizontal pleiotropy. Collectively, these results support the unidirectionality of the observed association—that is, CKAP2 levels may influence LUSC risk, rather than the reverse—and effectively rule out reverse causation as an alternative explanation for our primary findings.

Despite the limited causal evidence, the biological context of CKAP2 provides a plausible rationale for further investigation. CKAP2 is a microtubule-associated protein that is highly expressed during the G₂/M phase of the cell cycle and plays a critical role in maintaining spindle assembly checkpoint integrity and chromosomal stability. Overexpression of CKAP2 could disrupt the spindle assembly checkpoint, leading to genomic instability and aneuploidy [29, 30]. Previous research in ovarian cancer has shown that CKAP2 promotes cell proliferation and tumorigenesis through the FAK-ERK2 signaling pathway [31]. Given that FAK is highly expressed in LUSC and correlates with poor prognosis [32], it is plausible that CKAP2 facilitates LUSC tumorigenesis through similar mechanisms, such as activation of the MAPK pathway.

The LUSC-specific nature of the primary association is noteworthy, as LUSC pathogenesis is distinct from that of LUAD and SCLC. LUSC is often driven by recurrent genetic alterations, including TP53 mutations (occurring in approximately 59.2% of cases) and activation of the phosphatidylinositol 3-kinase-protein kinase B (PI3K/AKT) signaling pathway (observed in 47% of cases), with genomic instability manifested through chromosomal aberrations and centrosome amplification [33]. Importantly, our study identified a positive correlation between CKAP2 and TP53 expression in lung cancer, consistent with established molecular mechanisms. CKAP2 is a direct transcriptional target of p53; upon activation under cellular stress, p53 transcriptionally upregulates CKAP2, which subsequently contributes to cell cycle regulation, maintenance of genomic stability, and promotion of apoptosis [11]. While these correlations are consistent with the known p53–CKAP2 regulatory axis and with the high frequency of TP53 mutations in LUSC (~ 75%) [11], they do not, and cannot, serve as functional validation of the MR-inferred causal relationship. Correlation at the transcriptomic or proteomic level may reflect secondary changes accompanying tumor development rather than a direct causative pathway, and these descriptive analyses were not designed to test causality.

The downstream bioinformatic explorations—including differential expression analysis, ROC curve evaluation, immune infiltration correlation, co-expression network construction, and drug sensitivity profiling—were conducted with the explicit aim of generating exploratory hypotheses rather than providing confirmatory mechanistic evidence. Our analyses revealed that CKAP2 mRNA and protein levels were significantly elevated in LUSC tumor tissues compared to adjacent normal tissues, with robust diagnostic potential (AUC = 0.978). Functional enrichment analysis revealed that genes positively correlated with CKAP2 were predominantly enriched in cell cycle and mitosis-related processes, while negatively correlated genes were primarily associated with B cell-mediated immune activation pathways. Although previous observational studies have linked high CKAP2 expression to shorter overall survival in LUAD [34], the present MR analysis did not establish a causal relationship with LUAD. This discrepancy may arise from confounding biases common in observational study designs. LUAD pathogenesis is largely driven by mutations in EGFR, ALK, and KRAS, which constitutively activate downstream MAPK and PI3K-AKT signaling [35]. In contrast, SCLC pathogenesis lacks driver mutations in drug target genes such as EGFR and ALK [36], but instead features inactivation of TP53 and RB1 [37], along with MYC family amplification, Bcl-2 overexpression, and PI3K-AKT-mTOR pathway dysregulation—collectively driving SCLC’s aggressive phenotype with rapid doubling and early metastasis. The LUSC-specific CKAP2 association we observed may therefore reflect fundamental pathogenic differences among lung cancer subtypes. It is essential to reiterate that all of these correlative observations are descriptive and exploratory. They are valuable for generating testable hypotheses—for instance, that CKAP2 may influence LUSC progression through cell-cycle dysregulation and immune modulation, or that it could serve as a candidate biomarker for drug responsiveness—but they do not validate the causal pathway proposed by the MR analysis.

As a microtubule‑associated protein, elevated CKAP2 may predict improved responses to microtubule‑targeting agents such as paclitaxel in LUSC. In cervical cancer, Guo et al. showed that CKAP2 promotes tumor progression via NF‑κB signaling by increasing M2 macrophage infiltration, endothelial activity, angiogenesis, and vascular permeability, and that high CKAP2 expression correlates with paclitaxel sensitivity [12]. Consistent with these findings, our results reveal a negative correlation between CKAP2 expression and the half-maximal inhibitory concentration (IC₅₀ ) of paclitaxel. This suggests that tumors exhibiting elevated levels of CKAP2 may be more susceptible to chemotherapy based on paclitaxel. These observations support the potential role of CKAP2 as both a therapeutic target and a predictive biomarker for chemotherapy sensitivity in LUSC, thereby offering new avenues for personalized treatment strategies. Furthermore, this study illustrates that high CKAP2 expression modifies the tumor immune microenvironment in LUSC, characterized by reduced infiltration of B cells and CD4⁺ T cells. Elevated levels of CKAP2 have been associated with enhanced efficacy of Wee1 inhibitors and the chemotherapeutic agent MK-1775 (ID: 1179), suggesting that CKAP2 may serve as a predictive marker for treatment response and prognosis when using these agents. Furthermore, CKAP2 expression was higher in LUSC patients aged over 60 years, suggesting age‑related variation may influence therapeutic outcomes in older individuals. Reverse MR analysis found no causal effect of lung cancer on CKAP2 levels, ruling out reverse causality and supporting a directional role for CKAP2 in LUSC rather than a consequence of tumor development.

The discordant results between the discovery and replication analyses prompt us to explore several potential explanations. Several factors may account for the discrepancy of cross-Dataset Comparison analysis using an independent pQTL dataset (deCODE) : the deCODE pQTL dataset measured CKAP2 using a different proteomic platform (SomaScan) with distinct antibody specificity and dynamic range; the LUSC case count in the FinnGen replication cohort (*n* = 1510) was identical to the discovery cohort, thus power was not substantially reduced, but sample overlap cannot be definitively excluded; and population differences between the Sun et al. cohort (European-ancestry individuals from INTERVAL) and the deCODE cohort (Icelandic population) may contribute to genetic effect heterogeneity.

This study has several additional limitations. First, although all selected SNPs had F-statistics > 10, the modest sample size of the CKAP2 GWAS (*n* = 3301) may limit the strength of the instrumental variables. Second, while MR-PRESSO and MR-Egger tests did not detect significant horizontal pleiotropy, the limited number of instruments constrains the statistical power of these tests; therefore, the absence of detected pleiotropy should not be interpreted as definitive evidence that horizontal pleiotropy is absent. Third, the FinnGen dataset is predominantly of European ancestry, which limits the generalizability of our findings to other populations. Fourth, the downstream bioinformatic analyses (TCGA/CPTAC profiling, immune infiltration, and drug sensitivity correlation), although providing descriptive biological context, do not independently validate the causal relationship proposed by the MR analysis. Fifth, the relatively small number of LUSC cases (*n* = 1510) may reduce statistical power. Sixth, the molecular mechanisms linking CKAP2 to LUSC tumorigenesis were not experimentally examined in this study. Finally, we could not perform a parallel MR analysis for CKAP2L owing to the lack of suitable instruments in current datasets. Beyond these, as noted above, the primary MR finding did not survive multiple-testing correction and failed to replicate in the deCODE cohort, which collectively underscore the hypothesis-generating nature of this study.

## Conclusion

This bidirectional MR study provides suggestive genetic evidence for a nominal association between elevated CKAP2 protein levels and LUSC risk. The finding was supported by concordant CKAP2 upregulation in TCGA and CPTAC tumor tissues but was not replicated in an independent deCODE cohort. Given the preliminary nature of the causal evidence, the limited instruments, and the failed external cross-dataset comparison, CKAP2 should be viewed as a candidate for further biomarker validation rather than an established therapeutic target. Future studies with larger proteomic GWAS, diverse populations, and harmonized measurement platforms are needed to clarify whether CKAP2 plays a causal role in LUSC carcinogenesis.

## Supplementary Information

Supplementary material 1.

## Data Availability

The data utilized in this manuscript was obtained from publicly accessible resources. The summary data for CKAP2 were obtained from the IEU OpenGWAS project (https://opengwas.io/datasets/prot-a-564). Additionally, the summary data pertaining to total lung cancer, lung adenocarcinoma, lung squamous cell carcinoma, small cell lung cancer are available at https://storage.googleapis.com/finngen-public-data-r10/summary_stats/finngen_R10_C3_BRONCHUS_LUNG_EXALLC.gz, https://storage.googleapis.com/finngen-public-data-r10/summary_stats/finngen_R10_C3_NSCLC_ADENO_EXALLC.gz, https://storage.googleapis.com/finngen-public-data-r10/summary_stats/finngen_R10_C3_NSCLC_SQUAM_EXALLC.gz, and https://storage.googleapis.com/finngen-public-data-r10/summary_stats/finngen_R10_C3_SCLC_EXALLC.gz. respectively.
