# Supplementary material for: Genetic evidence for a potential role of cytoskeleton-associated protein 2 in lung squamous cell carcinoma: a bidirectional mendelian randomization study
Source: J Cardiothorac Surg. 2026 Jul 19;21:791. doi: 10.1186/s13019-026-04634-4 (PMC13663042; doi:10.1186/s13019-026-04634-4)
Supplement: Supplementary file 1 [file 13019_2026_4634_MOESM1_ESM.docx]

**Supplementary Table S1. Characteristics of the instrumental variables for CKAP2 protein levels**

| **SNP** | **Chromosome** | **Position** | **Effect allele** | **Other allele** | **EAF** | **Beta** | **SE** | ***P* value** | **R²** | **F-statistic** |
| --- | --- | --- | --- | --- | --- | --- | --- | --- | --- | --- |
| rs570618 | 1 | 196657064 | G | T | 0.611 | −0.167 | 0.025 | 4.57×10⁻¹¹ | 0.013 | 43.4 |
| rs142517401 | 3 | 66161137 | G | T | 0.115 | 0.222 | 0.040 | 3.24×10⁻⁸ | 0.009 | 30.5 |
| rs74480769 | 5 | 40972211 | G | A | 0.032 | −0.423 | 0.073 | 7.94×10⁻⁹ | 0.010 | 33.2 |
| rs2299257 | 7 | 94942765 | C | A | 0.383 | 0.154 | 0.026 | 2.24×10⁻⁹ | 0.011 | 35.8 |
| rs967645 | 17 | 26713970 | T | C | 0.509 | 0.176 | 0.024 | 5.13×10⁻¹³ | 0.016 | 52.4 |
| rs1065853 | 19 | 45413233 | T | G | 0.078 | 0.598 | 0.045 | 1.10×10⁻³⁹ | 0.050 | 173.9 |

**Notes:** All SNPs met genome-wide significance (*P* < 5×10⁻⁸) and linkage disequilibrium clumping criteria (R² < 0.001, 10000 kb). F-statistics > 10 indicate adequate instrument strength. EAF, effect allele frequency; Beta, effect size; SE, standard error; R², proportion of variance explained.

**Supplementary Figure S1.** Analysis of CKAP2 mRNA expression levels across different sex and pathological stages of LUSC. (A) CKAP2 expression by gender. (B) CKAP2 expression by pathological stage. CKAP2, cytoskeleton-associated protein 2; LUSC, lung squamous cell carcinoma.

### **Supplementary Table S2. Top 20 genes positively and negatively correlated with CKAP2 mRNA expression in LUSC (TCGA cohort)**

| Rank | Positively_Correlated_Gene | Negatively_Correlated_Gene |
| --- | --- | --- |
| 1 | BORA | CHRDL2 |
| 2 | SKA3 | CAPN8 |
| 3 | DIAPH3 | CPNE5 |
| 4 | RFC3 | IGLL5 |
| 5 | NUDT15 | CD79A |
| 6 | ECT2 | CD19 |
| 7 | SUCLA2 | C11orf96 |
| 8 | SLC25A15 | SLC9A3R2 |
| 9 | KPNA3 | CD79B |
| 10 | NUFIP1 | CCDC69 |
| 11 | SMC2 | MEI1 |
| 12 | LIN9 | JSRP1 |
| 13 | RACGAP1 | VASN |
| 14 | RMI1 | RRAS |
| 15 | HMGB1 | IGLL1 |
| 16 | DTL | P2RX1 |
| 17 | BUB1 | PTGDS |
| 18 | SPC25 | SOD3 |
| 19 | CKAP2L | CRIP1 |
| 20 | KIF11 | TNFRSF13B |

**Notes:** Positively correlated genes (co-expressed) and negatively correlated genes (antagonistically expressed) with CKAP2 were identified based on Pearson correlation analysis in the TCGA-LUSC cohort. CKAP2, cytoskeleton-associated protein 2; LUSC, lung squamous cell carcinoma; TCGA, The Cancer Genome Atlas

| Exposure | Outcome | Method | nSNP | Odds Ratio | 95% Confidence Interval | *P*-value |
| --- | --- | --- | --- | --- | --- | --- |
| CKAP2 (deCODE) | LUSC | Inverse variance weighted | 14 | 0.875 | 0.598-1.281 | 0.493 |
| CKAP2 (deCODE) | LUSC | MR Egger | 14 | 0.988 | 0.357-2.733 | 0.982 |
| CKAP2 (deCODE) | LUSC | Weighted median | 14 | 1.017 | 0.667-1.549 | 0.939 |

**Supplementary Table S3 Replication of the forward MR analysis for CKAP2 on LUSC risk using the deCODE pQTL dataset**

**Notes:** deCODE pQTL data from Ferkingstad et al. (Nat Genet, 2021) [19]; LUSC outcome data from FinnGen R10 [18]. MR, Mendelian randomization; CKAP2, cytoskeleton-associated protein 2; LUSC, lung squamous cell carcinoma; nSNP, number of single nucleotide polymorphisms.
